# Visualisation of Data Envelopment Analysis in primary health services

**DOI:** 10.1007/s10729-025-09702-0

**Published:** 2025-05-02

**Authors:** Ane Elixabete Ripoll-Zarraga, José Luis Franco Miguel, Carmen Fullana Belda

**Affiliations:** 1 Department of Telecommunications and Systems Engineering, Universitat Autònoma de Barcelona, 08193 Bellaterra, Spain; 2 Financial Management Department, Universidad Pontificia Comillas, 28015 Madrid, Spain

**Keywords:** DEA Visualisation, Multivariate Statistical Analysis, Primary Health Care, Health Specialisation, Extreme Cases, Efficiency

## Abstract

Benchmark efficiency analysis in public health typically focuses on hospitals rather than primary care providers. Data Envelopment Analysis (DEA) is widely used to assess resource efficiency among decision-making units (DMUs). However, traditional DEA struggles to differentiate between efficient units and is sensitive to the selection of inputs and outputs. Methods like super-efficiency and cross-efficiency address some of these limitations but often exclude outliers and may overlook efficiency related to specialisation. DEA Visualisation integrates DEA with multivariate statistical methods allowing for the identification of inefficiency sources and specialisation patterns without losing discriminatory power or removing extreme cases from the sample. This study analyses 82 public primary health centres in Madrid serving senior citizens in 2018. The findings reveal inefficiencies such as a preference for prescribing specific rather than generic drugs, increasing public health costs. Additionally, two extreme cases (outliers or mavericks) were identified as having high infrastructure costs and disproportionate staffing. Redistributing patients from overcrowded centres could enhance efficiency, while centres focused on preventive care showed greater cost-effectiveness, particularly in reducing prescription costs.

## Highlights

- DEA Visualisation helps reveal intrinsic characteristics of the data that are not immediately visible through facie evidence, such as specialisation.
- DEA Visualisation identifies sources of inefficiency, providing a basis for benchmarking and supporting managerial decision-making.
- DEA Visualisation detects outliers (or mavericks) without removing them from the sample, ensuring a more comprehensive analysis.
- The study reveals that some health centres are oriented towards preventive medicine through nursing care, reflecting a shift from curative to preventive practices.
- The study confirms that some health centres operate with overcapacity (idle resources), underutilising medical staff and infrastructure resources.
- There is evidence that certain centres prescribe specific (branded) drugs rather than generic, increasing costs for the regional health system.
- Prescribing practices involving specific drugs should be further investigated to determine whether economic incentives or other factors are influencing these prescription choices.

## Introduction

Primary care is essential to confront exceptional situations such as the spread of the pandemic caused by COVID-19, which began in the first quarter of 2020 becoming an international public health crisis[1]. The primary care future seems to be heading towards a model with greater integration between levels of care, which faces main challenges, such as the increase in technological costs, the pressure on health care and chronicity. While telehealth consultations have emerged to address hospital congestion, optimising staff allocation remains crucial to prevent overload and ensure diagnosis and treatment effectiveness, even remotely. Cultural backgrounds challenge adopting technological advancements in healthcare. This trend is evident in healthcare systems worldwide, such as the United Kingdom, Switzerland, New Zealand, and the United States [2]

Health decentralisation aims to reduce inequalities and improve accessibility. In the UK, the 1999 reform divided the National Health Service (NHS) into separate systems for England, Scotland, Wales, and Northern Ireland, shifting from the centralised model in hospitals established in 1948. Local authorities have limited control, while England's Integrated Care Systems (ICSs), introduced in 2022, focus on prevention to reduce disparities. In New Zealand, reforms since the 1980s created 14 Area Health Boards, later replaced by Regional Health Authorities to separate funding and service provision. Spain decentralised healthcare from 1984 to 2002, granting management to Autonomous Communities [3], with later reforms such as the 2007–2012 Strategic Framework improving primary care and professional training [4].

Since health management decentralisation, interest in hospital resource management efficiency has grown in supporting individual over centralised forms, as shown in benchmarking studies. With public health being costly, policymakers must ensure efficient resource allocation and avoid underutilisation. Efficiency estimation methods are classified as parametric, like Stochastic Frontier Analysis (SFA) (e.g., energy [5], airports [6]), and non-parametric, like Data Envelopment Analysis (DEA). SFA is less common due to its need for a specification form, including in healthcare [7, 8]. Few studies use DEA in health, with some comparing SFA and bootstrap DEA regression for panel data based on explanatory variables rather than efficiency scores [9].

DEA has become increasingly popular for efficiency studies in public and private services. For a literature review, see Liu et al. [10] and Emrouznejad and Yang [11]. In healthcare, most DEA studies focus on hospital efficiency [12–14] rather than primary care. Early applications include Nunamaker [15] on nursing services, Sherman [16] on teaching hospitals, and Huang [17] on general practice. DEA was first used after the 1984 reform to assess Spanish primary care before decentralisation, highlighting persistent deficiencies [18]. Later studies analysed efficiency in the Autonomous Communities, concluding that healthcare system reforms improved efficiency [19, 20]. Violan Fors et al. [21] suggested aligning staff with demographic needs to enhance service quality, while Cordero Ferrera et al. [22] incorporated patient characteristics and quality indicators. A managerial attempt to integrate healthcare services according to population health [23] by transferring primary care management to hospital concessionaires failed due to bureaucratic rigidity and challenges in public–private collaboration, including infrastructure investments [24] [1]. Deidda et al. [25] evaluated primary care centres with telecommunication devices in the Basque region using a four-stage DEA approach, incorporating the mortality index as a proxy for socio-economic factors. However, the link between economic power and health remains complex. Carrillo and Jorge [26] applied DEA to rank Spanish regional health systems, though differences in regulations and ownership may affect results. Cordero et al. [27] used a conditional non-parametric approach to estimate primary care efficiency, considering patient characteristics and hospitalisation rates potentially addressing different production functions and including uncontrollable undesirable outputs.

A key limitation of DEA is its inability to provide direct managerial recommendations, requiring second-stage analysis [28]. DEA identifies inefficiencies but does not account for random errors [29] and is highly sensitive to input and output selection, which depends on the researcher [28]. Methods like bootstrapping [30], stochastic non-parametric frontier models [31], and efficiency partial frontiers [32] address statistical limitations. Second-stage analysis often uses the Malmquist Index or regression to incorporate external factors, but these methods can be complex, reducing managerial applicability. Industry concerns focus on data reliability, yet researchers often use available data without verifying its accuracy, such as capital measures influenced by accounting policies [33]. Inputs and outputs should best represent the production function, as different variables can alter efficiency scores. Public services involve asymmetric information between taxpayers and providers, with access often shaped by political interests and legal factors. In the UK, hospital reports from the 1980s primarily used cost indicators [34], while performance measures have since expanded—e.g., Smith [35] includes resource provision and quality—empirical studies rarely scrutinise the chosen performance variables.

From an industrial perspective, benchmarking compares peers with similar activities, typically by size rather than the entire industry. In contrast, DEA compares all units, even those producing different services. Studies show that specialisation improves efficiency in healthcare [36], higher education [37], air transport [38], and ports [39]. DEA studies on primary care reveal diverse empirical findings, aiming to support managerial decisions but falling short of capturing the complexity of primary care outcomes [12]. Most are cross-sectional due to DEA's limited dynamism, and few have refined model specifications, particularly in selecting inputs and outputs [12]. DEA models must be carefully controlled to ensure accurate interpretation, particularly in primary care, which serves as the main entry point to the healthcare system [12, 40]. Variables (data) and benchmarking results (efficiency scores) should be critically assessed to determine their reliability for policy and managerial recommendations. A thorough analysis of operations and efficiency is needed for robust results.

Serrano-Cinca and Mar-Molinero [41] proposed embedding inputs and outputs selection (DEA specification) within a multivariate analysis framework—DEA Visualisation—which ranks fully efficient DMUs and highlights intrinsic characteristics. A key aspect is reducing data dimensionality using factor analysis to disclose underlying relationships between variables in efficiency estimation [42]. DEA Visualisation mitigates model misspecifications, identifies data characteristics, and detects performance peculiarities linked to specialisation. All units are benchmarked against each other, revealing common practices across possible DEA specifications. This method has been applied to internet providers [43], finance [44], higher education [45], airports [46], and water provision [47]. The results show inefficient sources according to all possible DEA specifications. To our knowledge, this is the first application of DEA Visualisation in healthcare, specifically for general practitioners' surgeries and walk-in centres.

Other techniques for variable selection in DEA include the Least Absolute Shrinkage and Selection Operator (LASSO) [48], incorporating non-discretionary inputs [49], and relaxing convexity constraints [50]. However, our study focuses on the underlying relationships between DEA specifications and observed data. Principal Component Analysis in DEA (PCA-DEA) creates new variables as linear combinations of the originals, preserving as much variance as possible. Rather than replacing inputs and outputs, we aim to explain efficiency scores. DEA Visualisation helps identify inefficiencies in managerial decisions, detects outliers without excluding them, and links efficiency to specialisation. By integrating DEA with multivariate analysis, we uncover behavioural patterns in DMUs, revealing the underlying meaning and characteristics of the data.

The paper is organised as follows. Section 2 describes the methodology used in the study. Section 3 presents the data. Section 4 provides the analysis and interpretation of results, along with a discussion on further insights into DEA Visualisation. Section 5 concludes with a summary of findings and suggestions for future research.

## Methodology

Charnes et al. [51] introduced Data Envelopment Analysis (DEA), later extended by Banker et al. [52], to assess the efficiency of homogeneous DMUs through benchmarking. DEA determines the path an inefficient unit must take to reach the efficiency frontier, using either radial [53, 54] or non-radial approaches [55, 56].

However, DEA has limitations [28], particularly in explaining inefficiencies. It often requires second-stage analysis with external factors. Popular techniques include truncated regression (excluding frontier units) and bootstrapping to strengthen confidence intervals. Yet, these external variables—chosen before analysis—lack integration with the internal production process, which remains a 'black box' affecting technical and scale efficiency.

DEA Visualisation addresses inefficiencies without relying on uncontrollable external factors, ensuring that inefficiency causes are assessed within the DMU operational context and efficiency improvements are more actionable.

Following Banker et al. [52], we use an output-oriented radial DEA model with variable returns to scale (VRS), imposing the restriction ∑λ = 1 to estimate efficiencies. As public health centres operate within budget constraints, primary care providers must maximise services with available resources. Inputs are limited by the assigned population, i.e., an external factor beyond management control. However, staffing levels (medical and non-medical) depend on the number of residents in an area. The model aims to explain why centres serving similar population sizes achieve different output levels.

$$Max\,\, \phi\,\, s.t.$$

1 $$\phi {y}_{i} \le Y\lambda$$

$$X\lambda \le {x}_{i}$$

$$\lambda \ge 0$$

$$\sum \lambda =1$$

The output-oriented DEA model determines how each unit can maximise production (outputs) without exceeding its available resources (inputs), with efficiency scores given by 1/ϕ.

Following Serrano-Cinca and Mar-Molinero [41], the two-way table—DEA specifications by DMUs— are analysed using multivariate statistical techniques, i.e., factor analysis, property fitting, and cluster analysis. Factor and cluster analysis identifies similarities, while property fitting examines proximities based on underlying characteristics.

Factor analysis reduces the number of DEA specifications by grouping those with similar efficiency scores, ensuring high within-group correlation but low correlation with other factors. It also helps discriminate between models with greater explanatory power, excluding one-input and one-output specifications. Property fitting maps DMUs and DEA specifications in a multidimensional space, revealing relevant input–output combinations and uncovering implicit data patterns not directly observed. It enhances the interpretation of efficiency dimensions and sources of inefficiency. Finally, cluster analysis groups DMUs with similar production functions based on these dimensions. For applications of this approach, see Gutierrez-Nieto et al. [57] and, for panel data, Ripoll-Zarraga et al. [58].

## Data description

DEA studies in primary healthcare typically classify inputs into three main categories: labour, capital, and consumable resources (e.g., medication usage), measured in physical units or monetary terms. Labour is often assessed by working hours or staff count by skill level. Outputs generally reflect centre activities, such as patient visits or tests performed. While quality is a crucial outcome, it is challenging to measure reliably.

Based on the literature review and data availability, four inputs and three outputs were selected for the DEA model (see Table 1). Inputs are labelled with letters, and outputs with numbers. The analysis focuses on 82 public primary healthcare centres in Madrid, Spain, for 2018—pre-COVID-19. The sample includes centres serving at least 5,000 citizens aged 65 and older, as younger populations have different healthcare needs. No centre in the study serves 15,000 or more residents. Each centre is assigned an acronym for identification (see Appendix 1, Table 6).

**Table 1:** Inputs and Outputs in the DEA Specifications

Inputs	Outputs
A Doctors	1 Visits
B Nurses	2 Prescription Cost
C Admin Staff	3 Vaccines
D Infrastructure Costs	

On the input side, the number of doctors, nurses, and administrative staff is measured in full-time or full-time equivalent terms. Interviews with different centres confirmed that part-time staff is rare since full-time employees have no incentive to work in private facilities. Infrastructure costs represent fixed expenses, including utilities, cleaning, maintenance, and rent. These costs are a depreciation proxy due to the unavailability of accounting policy data or property ownership details (i.e., whether premises are rented or owned). For outputs, the models include the number of patient visits, the average annual prescription cost per patient aged 65 and older in Madrid province, and the number of vaccines administered.

Visits refer to the total number of medical appointments scheduled and attended annually. This measure does not account for no-shows (an undesirable output). A single patient may have multiple appointments in the same year, including recurrent visits. These visits can involve general practitioners (GPs), paediatricians, nurses, or a combination within the same appointment. Other medical services, such as tests and treatments, are primarily provided in hospitals. While all centres have GPs and nurses, paediatric services may be unavailable in every facility if another centre within the same catchment area offers this service.

The unitary prescription cost represents the expenditure incurred by the publicly funded health centre, subject to restrictions based on patients’ circumstances. Prescription drugs fall into two categories: generic and branded drugs. Generic medicines are produced by multiple laboratories and are significantly cheaper. These are often fully or partially covered by the national social security system, depending on factors, such as age, employment status, and social benefits. Branded (patent-protected) drugs are more expensive and not always covered by public funding.

Similarly to other industries where costs are treated as inputs (e.g., air transport, where expenses generate revenue), studies in primary healthcare often use prescription costs as an input. In this analysis, the goal is not to evaluate resource allocation efficiency but rather cost-effectiveness—specifically, how well a centre can reduce prescription costs. Since doctors have decision-making power in prescribing generic versus branded drugs, prescription costs reflect medical activity outcomes that may be constrained by resource availability (e.g., the number of doctors).

The number of vaccines is the total number for flu and paediatrics. Other types of vaccines are injected at the hospitals.

The model calibration initially considered the type of prescription, assuming that centres prescribing more generics would be more efficient due to lower costs. However, since all centres had a similar percentage of generic prescriptions (50–60% in 2018), this variable lacked discriminatory power for visualisation and was excluded. Instead, the unitary prescription cost was used, as lower costs imply greater efficiency by reducing public expenditure.

Additionally, patient satisfaction was included as a desirable output. While DEA applications using percentage-based data (ratios) are relatively new [59], satisfaction data showed low variability (89% average across 262 centres in 2018), limiting its usefulness for differentiation. In private healthcare, dissatisfied patients often switch providers, whereas public healthcare allocation depends on geographic location and population. Ripoll-Zarraga and Mar-Molinero [46] addressed a similar issue by using the percentage of on-time flights to replace delays (an undesirable output), ultimately identifying a factor related to punctuality and passenger satisfaction.

There are no published data on actual annual revenue per centre. In Spain, public healthcare operates under universal coverage, meaning income is estimated based on the allocated budget yearly, which is derived from accrued expenses from the previous period. This includes salaries, infrastructure, and prescription costs, potentially leading to endogeneity issues.

Table 2 presents the relevant statistics, with all data deflated using Spain’s 2015 GDP deflator [60].

**Table 2:** Descriptive Statistics Deflated GDP (2018)

Variable	Observations	Mean	Std. Deviation	Min	Max
Doctors (Number)	82	18.94	7.53	3	41
Nurses (Number)	82	14.13	5.74	3	31
Admin Staff (Number)	82	12.00	4.42	2	25
Infrastructure Costs (€)	82	536,562.56	139,001.92	319,003.08	875,505.81
Visits (Number)	82	231,561.51	62,321.49	120,320.00	414,943.00
Prescription Cost (€/unit)	82	154.80	31.00	80.31	226.94
Vaccines (Number)	82	8,877.03	2,229.32	5,010.36	15,739.53

Some combinations of inputs and outputs are logically inconsistent and will be excluded from the factorial analysis. For example: doctors (A) prescribe drugs (2), but nurses (B) and admin staff (C) do not, making combinations like B2 or C2 unrealistic. Prescription cost (2) should be linked to visits (1) and not assessed alone or with vaccines (3) without patient interaction. Vaccines (3) should also be linked to visits (1) since a patient must be present to receive a vaccine. The factorial analysis algorithm excludes these non-sensical combinations. The variables generate 15 input combinations and seven output combinations. This results in 105 distinct DEA specifications (Appendix 1, Table 5). A subset of health centres and efficiency results for 13 DEA specifications is illustrated in Table 7 (Appendix 2).

## Results

The traditional DEA model, incorporating all inputs (A: Doctors, B: Nurses, C: Admin Staff, D: Infrastructure Costs) and all outputs (1: Visits, 2: Prescription Cost, 3: Vaccines), identifies 14 efficient centres. The most inefficient centres based on efficiency scores are ELOY (141.35%), VILO (139.87%), and INFA (138.83%). These centres exhibit inefficiencies exceeding 130%, suggesting significant room for improvement in resource utilisation relative to their peers. A detailed discussion of key findings and a comprehensive set of results are available in the appendices.

### Reducing the Dimensionality of the Data: Factor Analysis

Factor analysis reduces the dimensionality of the DEA specifications while retaining meaningful information. In small samples, intrinsic data characteristics can be assessed directly, but for large datasets, multivariate statistics analysis enables visual interpretation. The initial matrix consists of 82 health centres (observations) and 105 DEA specifications (variables) [41]. The first stage applies factor analysis to group specifications that produce similar efficiency scores across observations while remaining independent of other factors. This transforms the initial matrix into a new matrix with centres in rows and extracted dimensions (factors) in columns.

An initial unrotated factor analysis identified multiple factors with eigenvalues above one (Kaiser criterion). A second analysis, applying varimax rotation, improved visual interpretation, allowing for clearer identification of dimensions. The Kaiser criterion identified seven factors, aligning with Jolliffe’s criterion [61], which uses a less strict threshold (eigenvalue > 0.70). Notably, no factors had eigenvalues between 0.70 and 1. The results are presented in Table 3.

**Table 3:** Factor Analysis. Variance explained

Component	Initial Solution			Rotated Solution		
	Eigenvalue	% of Variance	Cumulative %	Eigenvalue	% of Variance	Cumulative %
**1**	59.63	60.85	60.85	34.21	34.91	34.91
**2**	19.08	19.47	80.32	24.29	24.78	59.69
**3**	10.00	10.20	90.52	23.53	24.01	83.70
**4**	3.21	3.27	93.79	6.48	6.61	90.31
**5**	2.01	2.05	95.84	3.72	3.79	94.10
**6**	1.45	1.48	97.32	2.80	2.86	96.96
**7**	1.16	1.18	98.50	1.51	1.54	98.50
**8**	0.44	0.45	98.95			

The first two components account for 80% of the data variability: factor 1 explains 61%, factor two 19%, and factor three 10%, while the remaining factors contribute marginal effects (between 0.4% and 3%). Despite their smaller impact, these factors may highlight outliers or mavericks [62], making them worth investigating. Notably, factor eight is not relevant in either solution. The first three factors collectively explain over 90% of the variability in the unrotated solution, indicating that the extracted components effectively summarize the intrinsic characteristics of the units analysed.

The factor loading matrix (Table 8, Appendix 3) provides meaning to each factor, with loadings below 0.4 removed to facilitate interpretation. Most DEA specifications within factor 1 exhibit strong positive correlations, typically interpreted as overall efficiency. However, in the rotated matrix, variability is distributed between factors 1 and 2. The component matrix reveals that DEA specifications involving only output 2 (Prescription Cost) have the highest loadings in factor 1, regardless of the inputs used (ranging from 0.93 for D2 to 0.98 for ABC2, AB2, AC2, BC2, A2, B2, C2). These results suggest that factor 1 represents efficiency in prescribing generic drugs at lower unitary cost. The highest loading corresponds to AC2 (0.9818), combining the number of doctors (A), admin staff (C) and prescription cost (2).

**Fig. 1:**
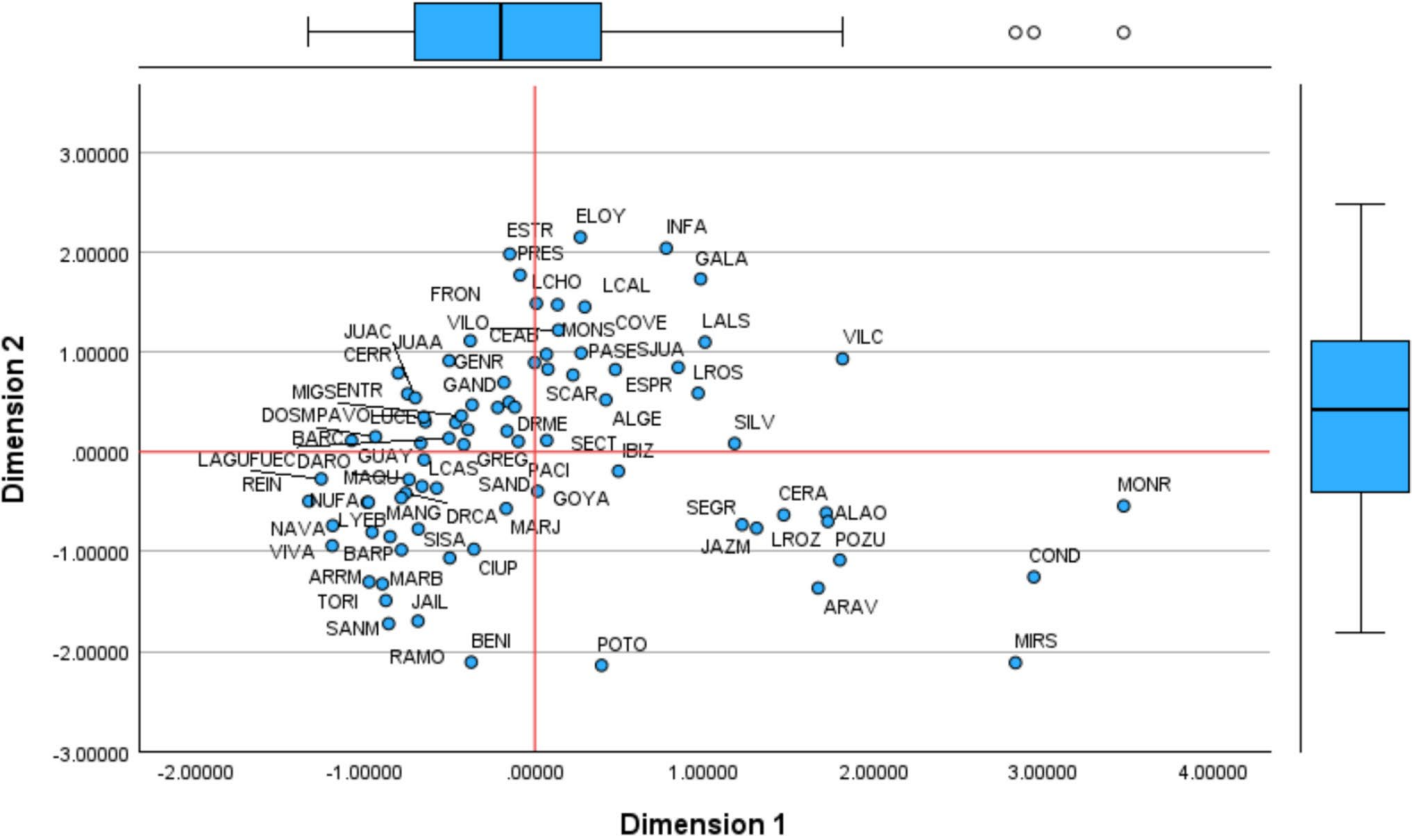
Common Map. Factors 1 and 2

**Fig. 2:**
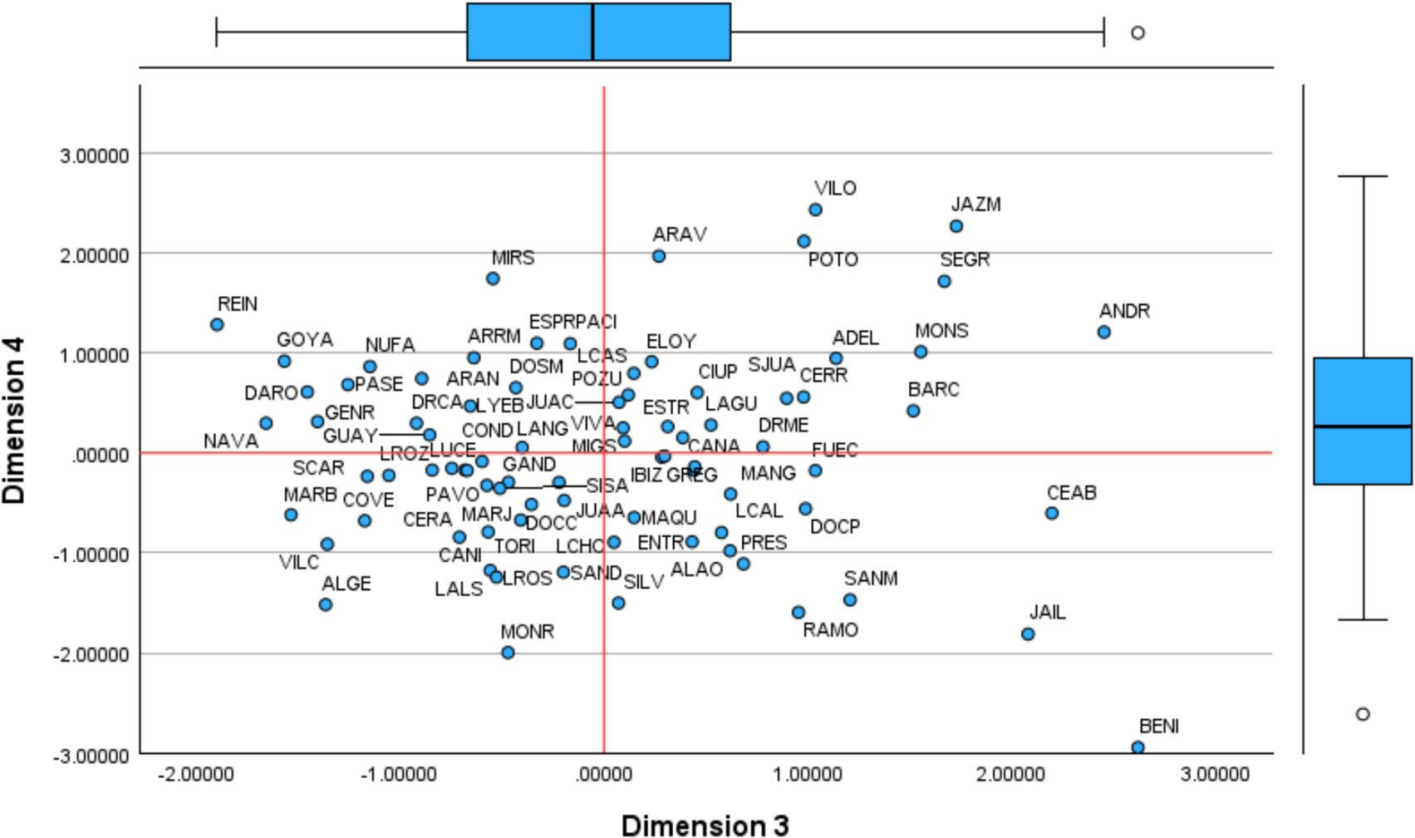
Common Map. Factors 3 and 4

For other factors, interpretations are less straightforward. The factor analysis provides the coordinates in the common map (multidimensional space), positioning each DMU in relation to each component (component matrix). Using Table 4, we map each health centre based on the first four components (Figs. 1 and 2). Additional figures are included in Appendices 5 (Figs. 8 and 9).

**Table 4:** Coordinates of Health Centres in the Common Space (Rotated Component Matrix)

	**Dim1**	**Dim2**	**Dim3**	**Dim4**	**Dim5**	**Dim6**	**Dim7**
ADEL			1.138	0.945		1.360	−0.857
ALAO	1.717	−0.613	0.618	−0.980	1.942		0.692
ALGE			−1.366	−1.518		−0.860	−0.578
ANDR		0.893	2.452	1.208	−0.906	0.929	0.789
ARAN	−0.647		−0.894	0.743			0.613
ARAV	1.670	−1.366		1.967	1.599	−1.713	
ARRM	−0.978	−1.304	−0.639	0.950	0.144	0.132	
BARC	−1.081		1.517		−2.570	−0.904	
BARP	−0.787	−0.983	−1.056				
BENI		−2.108	2.619	−2.947	0.669		1.477
CANA	−0.467					0.662	
CANI			−0.568	−0.791	0.880		
CEAB		0.976	2.197	−0.604	−0.608	1.046	−1.891
CERR	−0.806	0.789	0.979	0.558	−1.595	−0.657	−0.961
CERA	1.467	−0.633	−0.709	−0.843		0.567	−0.856
CIUP		−0.977		0.602			0.766
COVE		0.989	−1.174	−0.679	1.088	−0.614	−1.131
COND	2.942	−1.255	−0.598		−2.704		
DARO	−0.742		−1.456	0.609			
DOCC	−0.579			−0.517	0.935		
DOCP			0.988	−0.559			0.652
DOSM	−0.940			0.651	−0.845	−0.471	0.524
DRCA	−0.761		−0.920				1.231
DRME			0.780			0.888	
ELOY		2.149		0.911		0.889	
ENTR	−0.750	0.580		−0.890		−2.746	
ESPR	0.845	0.844		1.096		0.874	1.561
ESTR		1.980				−1.319	
FRON		1.112				−1.699	
FUEC	−0.507		1.036		−0.552		
GALA	0.977	1.731	−0.682			0.752	
GAND		0.694	−0.469			−0.412	0.521
GENR			−1.405				1.229
GOYA			−1.568	0.917			1.617
GREG	−0.394		0.283			0.542	
GUAY	−0.654		−0.855				1.291
IBIZ	0.492				0.756	0.619	0.842
INFA	0.774	2.039			0.795	0.608	
JAIL	−0.863	−1.723	2.080	−1.813	−1.058	1.008	−0.690
JAZM	1.307	−0.766	1.728	2.267	1.652	−1.097	
JUAA	−0.506	0.912		−0.476		−2.037	
JUAC	−0.705	0.540		0.505	−0.726		
LCHO		1.486		−0.894		−2.020	
LAGU	−1.260		0.524		−1.585	−1.062	
LCAL		1.451	0.576	−0.797		−0.759	
LROZ	1.727	−0.700	−0.672				
LALS	1.003	1.098	−0.558	−1.176	1.473		
LANG	−0.989	−0.508			−0.509		1.307
LCAS	−0.666			0.795		0.507	0.451
LROS	0.963	0.586		−1.243	0.835		0.934
LYEB	−0.854	−0.851	−0.656				1.935
LUCE	−0.673		−0.843			−0.816	
MANG	−0.960	−0.805	0.621		−0.643		−0.953
MAQU	−0.788			−0.647			1.646
MARB		−1.065	−1.535	−0.620		0.570	0.630
MARJ		−0.569	−0.575		0.960		−0.750
MIGS	−0.433	0.359				0.720	
MIRS	2.833	−2.114		1.743	−2.940	−1.789	−0.811
MONR	3.472			−1.997	−2.759	0.971	−0.752
MONS		1.473	1.554	1.010		1.270	1.790
NAVA	−1.195	−0.942	−1.658		0.965		
NUFA	−0.982		−1.148	0.862	−0.568		
PACI				1.091	0.582	0.795	
PASE		0.827	−1.256	0.682		1.221	0.568
PAVO	−0.655		−0.747			−1.142	
POTO		−2.140	0.981	2.116	1.235	−1.638	−0.807
POZU	1.798	−1.085		0.579	1.717	−0.939	
PRES		1.770	0.684	−1.110		−3.145	−0.985
RAMO	−0.690	−1.697	0.954	−1.595			0.662
REIN	−1.333		−1.898	1.282	0.861	1.544	−4.957
SAND				−1.193	0.507	−1.360	
SCAR		0.768	−1.161				
SJUA		0.823	0.896	0.545	0.656	1.443	
SANM	−0.879	−1.491	1.207	−1.470		0.796	−0.879
SISA	−0.688	−0.774	−0.511		0.576		−0.504
SECT				−0.140	0.762	1.034	−1.212
SEGR	1.222	−0.731	1.669	1.715	1.630	−0.687	
SILV	1.177			−1.502	1.991	0.592	
TORI	−0.899	−1.324		−0.673			0.901
VIVA	−1.193	−0.740			−0.705		−1.846
VILC	1.814	0.931	−1.357	−0.914	−2.120	2.054	
VILO		1.219	1.037	2.431		1.441	−0.808

Considering the location of the centres concerning dimension 1, Monterrozas (MONR), Condes de Barcelona (COND), and Mirasierra (MIRS) demonstrate the best performance. These centres incur the lowest unitary prescription costs —80.31, 89.16, and 91.29 euros per prescription, respectively. Among them, MONR is the most efficient centre, achieving the lowest unitary prescription cost overall. Conversely, centres such as Barcelona (BARC) and Las Aguilas (LAGU), are the most inefficient, with significantly higher costs of 199.63 and 226.94 euros per prescription, respectively. Reina Victoria (REIN) is positioned near these two centres but has a lower unitary cost (125.27). A comparison with INFA (125.28) shows similar prescription costs, yet INFA is located differently on the unitary value axis (x-axis). This positioning suggests that REIN may be an extreme case, to be analysed later. Factor 1 appears to highlight efficiency in prescribing generics, serving as an overall efficiency measure for cost-effectiveness in medication prescriptions.

From a managerial perspective, given that these centres provide public services, it is necessary to question why some prescribe more expensive medicines instead of generics. If centres prescribe costly drugs when generic alternatives are available, this may suggest hidden commissions received by doctors —i.e., economic incentives to favour certain laboratories over more affordable options. Figure 1 indicates that a significant number of centres may be engaging in this practice, as they are from 0 to the left side of the x-axis. Regarding dimension 2, centres mapped between 1 and 2 of the y-axis show high inefficiency in nursing activities. ELOY, ESTR, and INFA incur higher costs than RAMO, BENI, POTO or MIRS, while vaccinating fewer people. These centres could increase efficiency by vaccinating more patients or absorbing populations from other areas. However, this analysis does not account for additional nursing responsibilities such as assisting doctors, wound care, and patient monitoring. We name Factor 2 as the underuse of infrastructure in administering vaccines (i.e., by nurses).

Factors 3 and 4 reveal an extreme case of BENI. For dimension 3 BENI is nearby ANDR and CEAB. These three centres, with similar infrastructure costs, have a significant staff for the activity performed. BENI is one of the centres with more doctors and nurses and relatively lower activity than ELOY (near zero value on the x-axis), which operates with 10 fewer doctors, nine fewer nurses, and four fewer administrators. On the opposite side of the x-axis, NAVA or REIN have fewer employees but much higher visits and vaccines administrated. This factor reveals that BENI, along with other centres nearby (e.g., JAZM, SEGR, MONS, BARC), is inefficient regarding staff and has an excessive resource allocation for the activity performed. BENI was on frontier in the first DEA specification, accounting for all inputs and outputs (ABCD123). However, this does not reflect the reality, as it has excessive staff for the services provided, leading to underutilising infrastructure. We name Factor 3 as staff inefficiency. Regarding Factor 4, VILO and JAZM at the top and RAMO, JAIL, and BENI at the bottom have similar infrastructure costs and vaccination activities, yet the later generate more visits. It is evident that BENI has a significantly higher number of employees and appears isolated regarding dimensions 3 and 4. We name Factor 4 as infrastructure usage to generate visits (with doctors). Centres at the bottom of dimension 4 incur high infrastructure costs but provide fewer services than expected. It suggests an issue of excessive capacity, with some centres being too large for the population they serve.

The interpretation of factors is complex, as each centre exists within a 105-dimensional space based on the DEA specifications estimated. In some dimensions, centres are distributed across the axis, making it difficult to determine their true proximity. Although two centres may appear close in a lower-dimensional representation, this may not reflect their actual position in multidimensional space. To address this, we apply property fitting (ProFit) to evaluate the relative positioning of observations and DEA specifications, helping to undercover the underlying meaning of the dimensions.

### Identifying Sources of Inefficiency: Property Fitting

Following Schiffman et al*.* [63] and Gower and Hand [64], the Property Fitting approach allows mapping the DEA specifications and variables [65] in the same space (common map) to reveal the meaning of each factor. ProFit generates a vector for each DEA specification showing the direction towards the model specification changes. A regression is performed to draw each vector, one per each DEA specification, which is the independent variable, and the dependent variables are the extracted seven factors. The ProFit vectors are normalised to unitary length following

2 $${\beta }_{jl}^{*}=\frac{{\beta }_{jl}}{\sqrt[2]{{\sum }_{l=1}^{7}{\beta }_{l}^{2}}}$$

The ${\beta }_{jl}$ is the $l-$ th regression coefficient (Factor $l$) associated with each $j$ DEA specification (unstandardised coefficients). In total, 105 regressions (DEA specifications) will generate 105 standardised times seven betas, with the origin in the centre of coordinates. The length indicates the relevance of each dimension (extracted factor), such as when the standardised beta of a DEA specification has a unitary value ${(\beta }_{jl}^{*}=1)$ or is close to one, the related dimension is significant for interpretation. Whereas if the vector near the origin of coordinates, the dimension is irrelevant for definition. The normalised ProFit vectors (endpoints) are shown in Table 9 (Appendix 4).

**Fig. 3:**
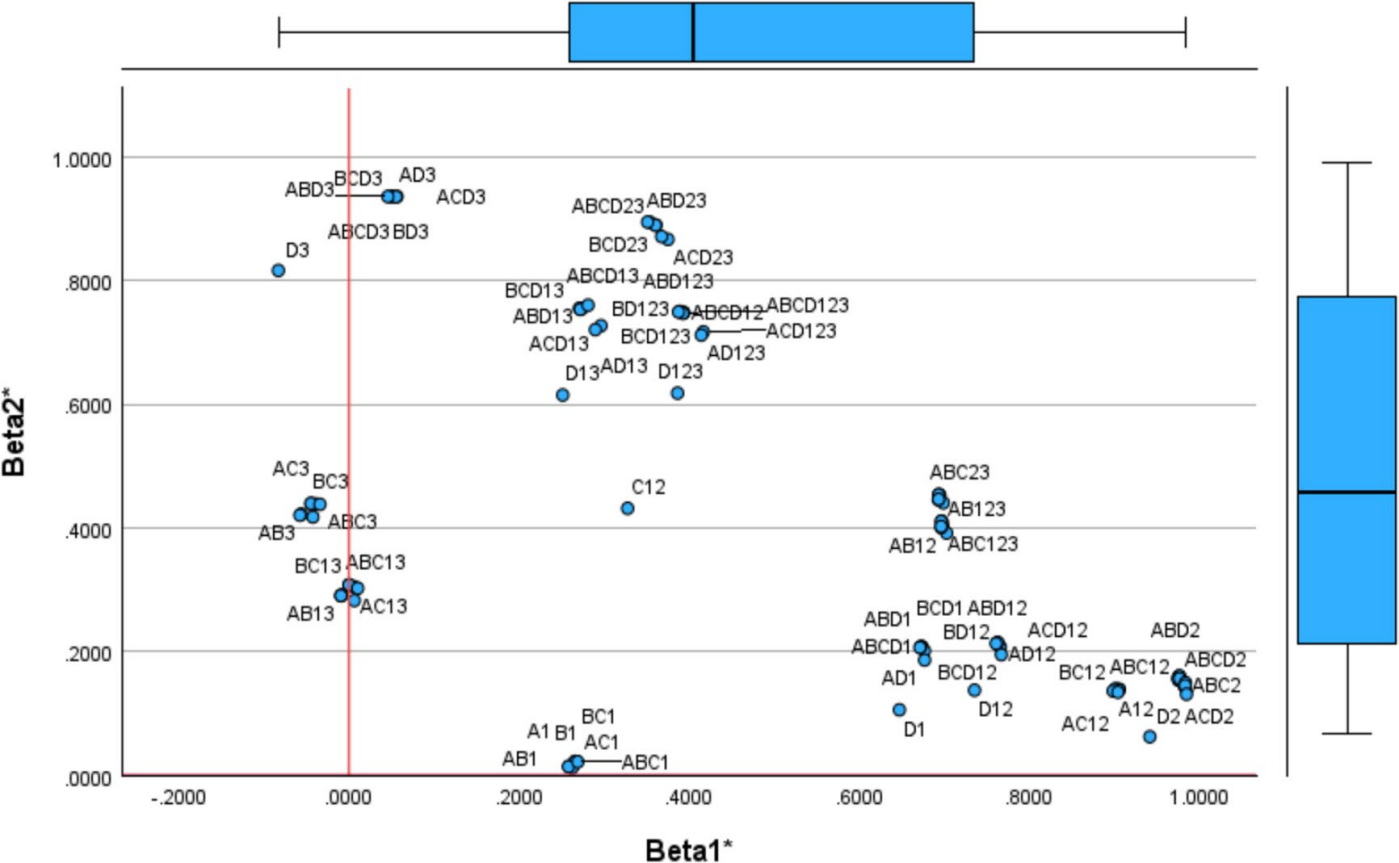
ProFit DEA specifications projection (Dimension 1 and Dimension 2)

**Fig. 4:**
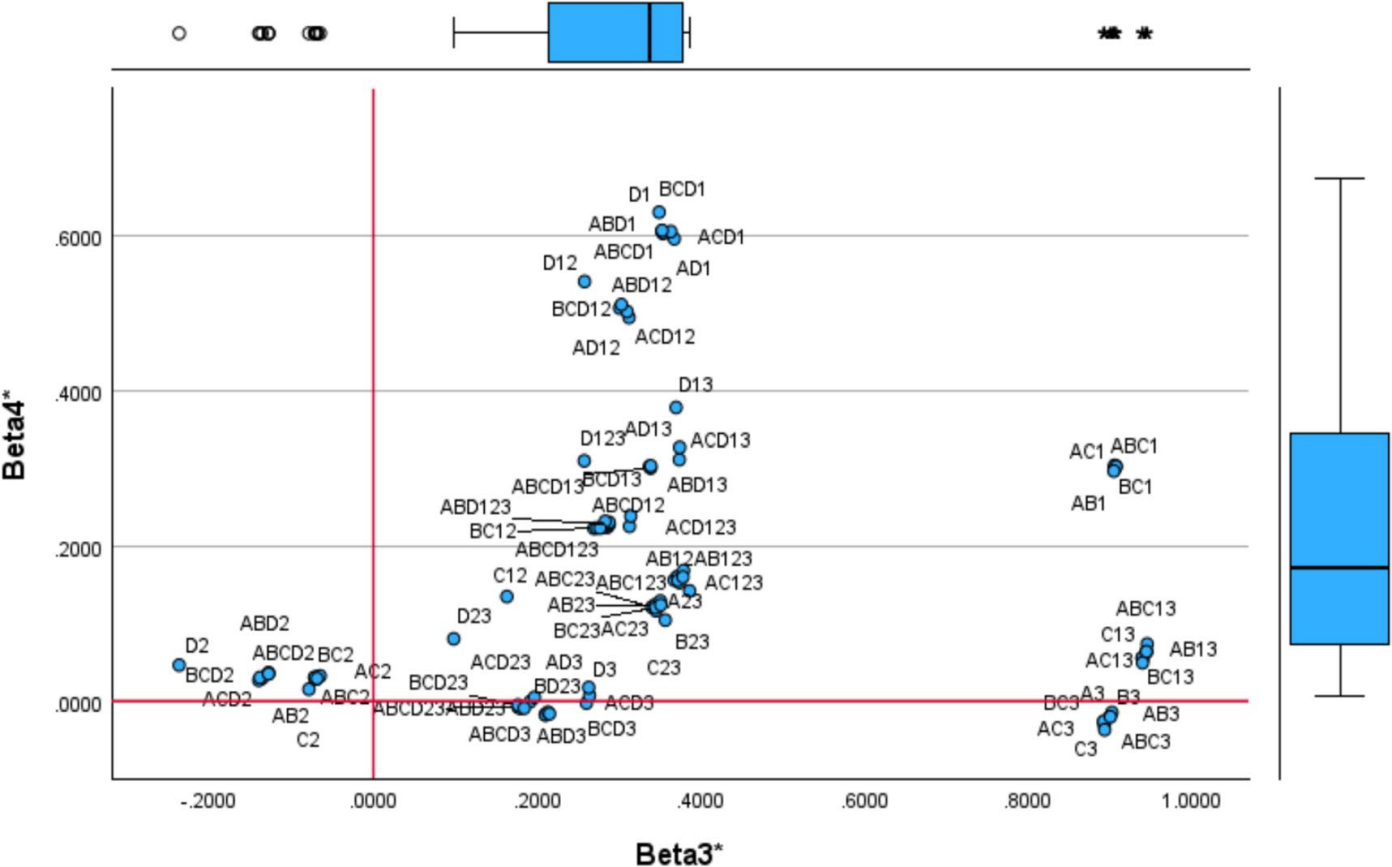
ProFit DEA specifications projection (Dimension 3 and Dimension 4)

Combining both matrices, the health centres in the common map (Table 4) and the ProFit vectors (Table 9), enhances the accuracy of factor interpretation by providing a more comprehensive understanding of the data structure. Figure 3 illustrates the location of DEA specifications according to factors 1 and 2, incorporating property fitting vectors, while Fig. 4 presents factors 3 and 4. The remaining figures are included in Appendix 6 (Figs. 10 and 11). Each DEA specification is a point in space. The proximity of a point (DEA specification) to the arrowhead indicates its relevance to the corresponding dimension.

**Fig. 5:**
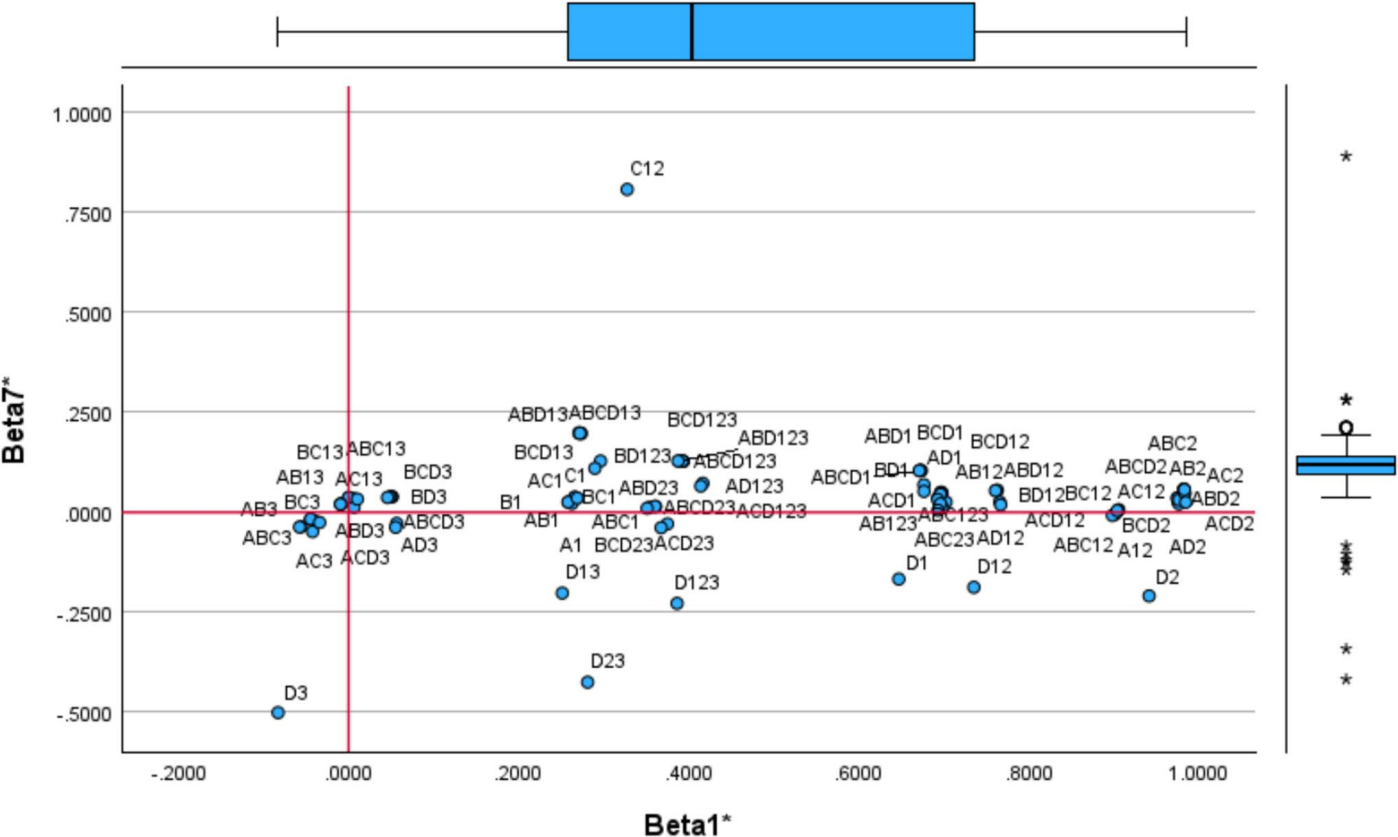
ProFit for Dimensions 1 and 7

The model specification positioned at the end of x-axis is ABCD2. Other input combinations with output 2 (Prescription Cost) are also concentrated at the end of the axis, reinforcing the interpretation of dimension 1 as the efficiency of centres in prescribing generic drugs. Dimension 2 features DEA specifications that are closer to the arrowhead, particularly those incorporating input D (Infrastructure Costs) and output 3 (Vaccines). This alignment confirms the previously identified interpretation of dimension 2 as the infrastructure usage in administering vaccines, specifically by nurses (Fig. 5).

Using ProFit, we identified dimension 3 as staff efficiency in providing services such as visits and vaccinations (AB13, BC13, ABC13) and dimension 4 as the use of infrastructure (D) to generate visits (1). Dimension 5 reflects the willingness (or reluctance) towards preventive medicine through nursing care (B) and vaccination (3). Centres on the right-hand side of Fig. 8 are more inefficient or reluctant to adopt this practice. For instance, MIRS, MONR and COND administer a relatively high number of vaccinations while maintaining low prescription costs, suggesting a focus on nursing care and alternative therapies despite higher operating costs. Conversely, SILV and ALAO, have lower vaccination rates and higher prescription costs.

Dimension 6 highlights the common input (D) used to generate outputs (1 and 3), representing technical efficiency in infrastructure use per visit, including vaccination. Centres at the top of dimension 6, such VILC, must increase visits to absorb infrastructure costs. Dimension 7 measures the efficiency of non-medical staff in handling visits and promoting generic drug prescriptions (C12). Centres at the bottom of dimension 7, such as BENI, GOYA and LYEB, operate efficiently, with fewer administrative staff while maintaining service levels comparable to centres with larger teams.

Dimension 7 also reveales an extreme case: REIN, the centre with the fewest employees but a high number of visits at a lower unitary prescription cost. REIN is efficient overall (ABCD123) and, apart from seven specifications relying solely on infrastructure (D), is technically efficient in all other models. While highly efficient in staffing (dimension 3, ProFit ABC13) and administrative functions (dimension 7, ProFit C12), REIN incurs excessive operating costs (dimension 4, ProFit D1) and high unitary prescription cost compared to similar-sized centres (dimension 1, ProFit ABCD2). This overcapacity suggests that redistributing staff could enhance activity levels—more visits and prescriptions—helping to absorb infrastructure costs. The low staff numbers may also indicate that doctors prescribe more expensive drugs s than generics, potentially to offset operational expenses. REIN prescription cost averages 125.27 euros per unit, comparable to or exceeding centres with more than three times its personnel.

### Identifying Common Practices Benchmark: Cluster Analysis

**Fig. 6:**
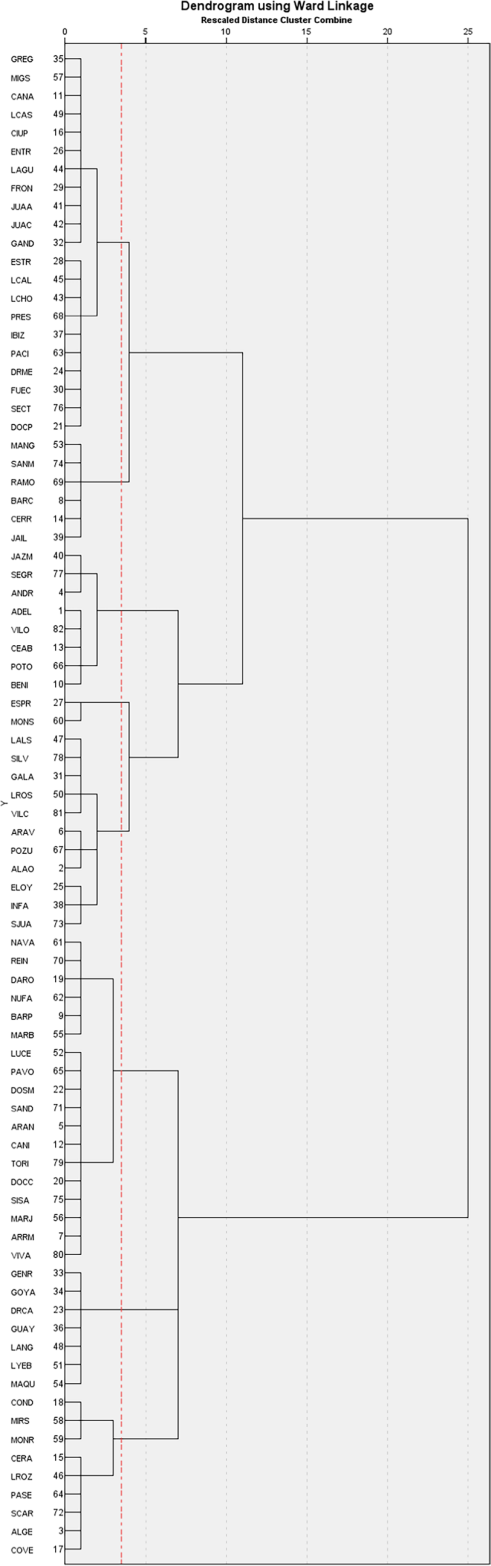
Dendrogram. Ward Method (Unstandardised)

**Fig. 7:**
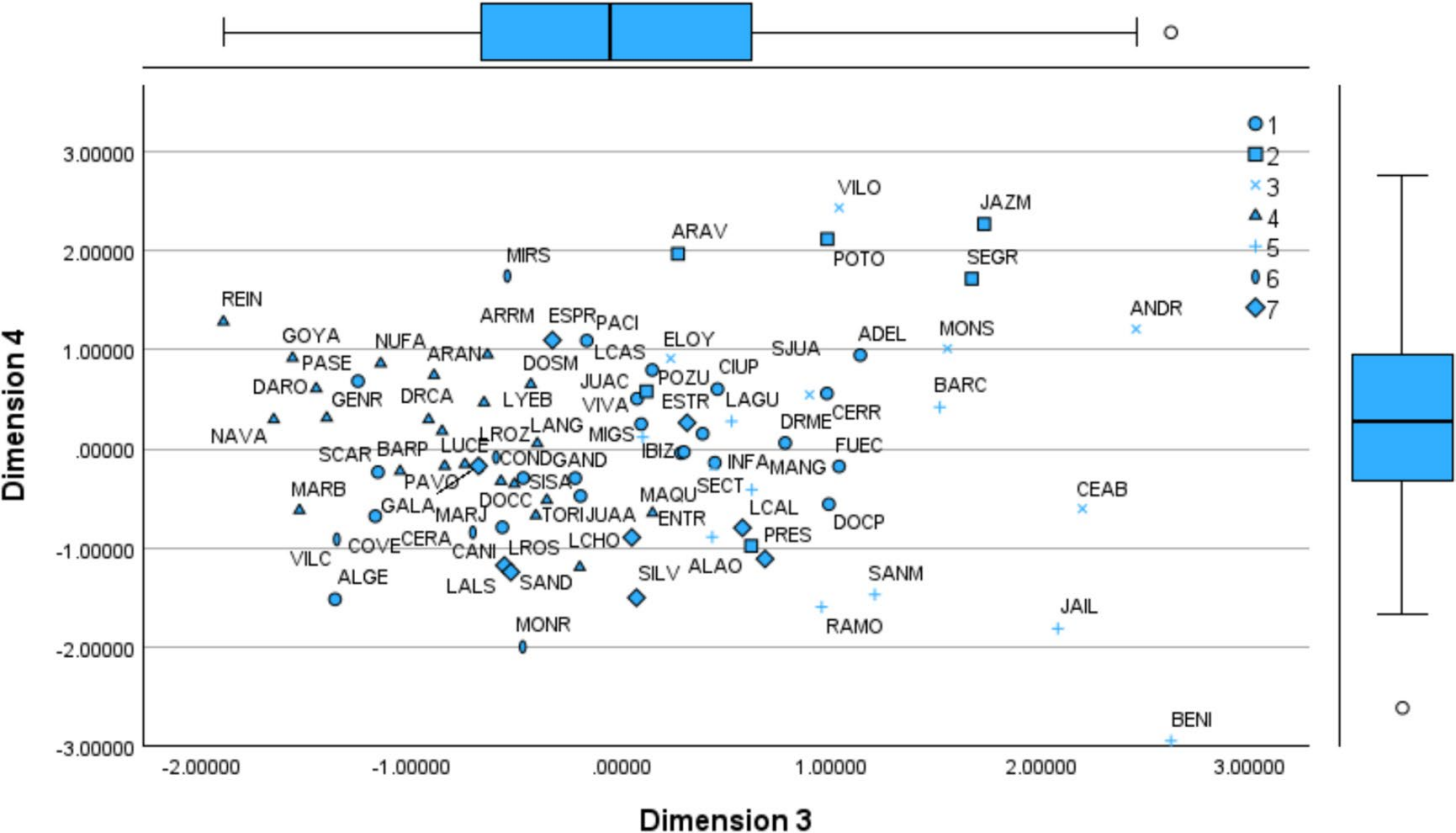
Common Map. Cluster Analysis (Factors 3 and 4)

**Fig. 8:**
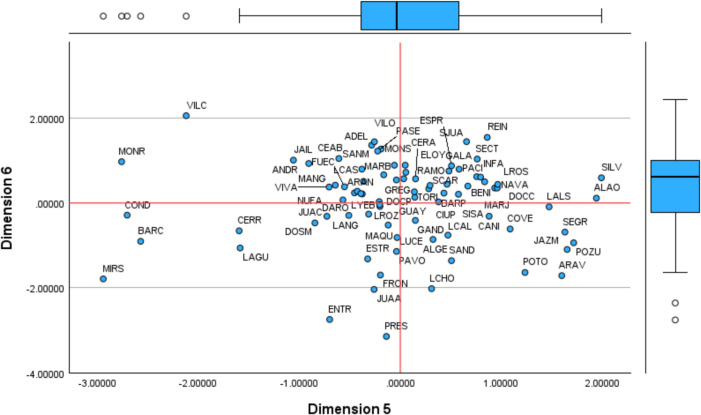
Common Map. Factors 5 and 6 (Rotated Solution)

We conduct an additional stage using cluster classification to group centres that operate similarly in terms of input usage and output generation. This approach allows us to identify outliers without excluding them from the sample. The goal of cluster analysis is to determine the ‘real’ competitors of each DMU, identifying which efficient unit should serve as benchmarks for inefficient ones to reach the frontier. These reference units operate under similar conditions or specialise in comparable activities. For example, centres specialised in nursing care should not attempt to emulate those that primarily assess patients and prescribe medications. We apply the Ward method to ensure maximum homogeneity within clusters while maintaining clear distinctions between them. The dendrogram suggests between eight to 14 clusters (see Fig. 6). For instance, JAZM, SEGR, and ANDR fall within the same cluster, as do COND, MIRS and MONR indicating shared operational characteristics. To simplify the analysis, we select seven clusters (Fig. 7).

We map the centres classified into their clusters according to the seven extracted factors. We show one figure for illustration purposes. For a further breakdown see Appendix 7 (Figs. 12 and 13).

**Fig. 9:**
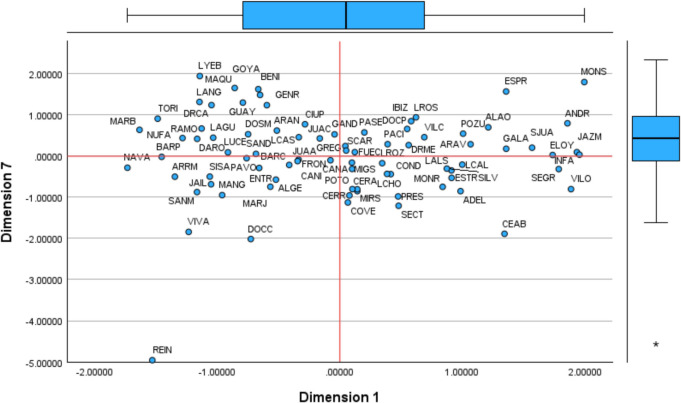
Common Map. Factors 1 and 7 (Rotated Solution)

**Fig. 10:**
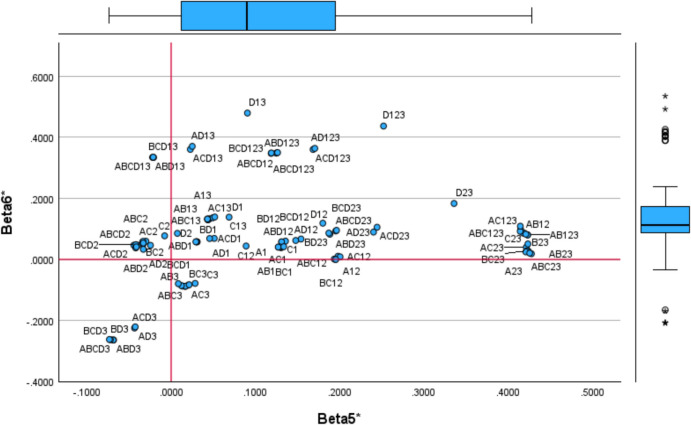
ProFit for Dimensions 5 and 6

**Fig. 11:**
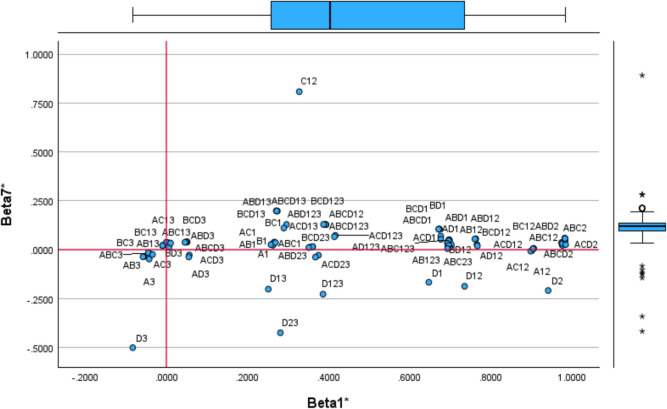
ProFit for Dimensions 1 and 7

**Fig. 12:**
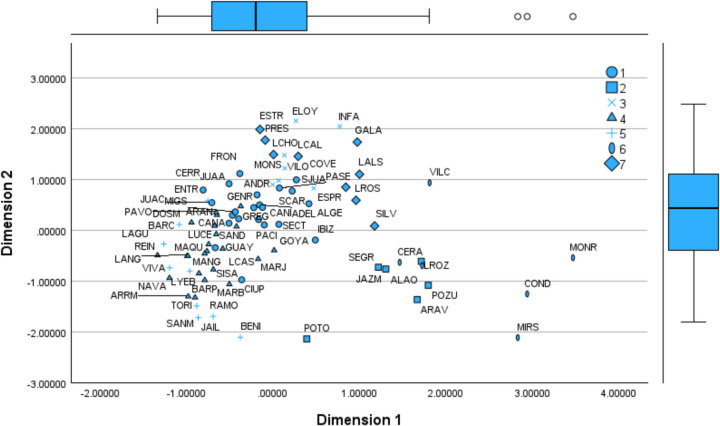
Common Map. Cluster Analysis (Factors 1 and 2)

**Fig. 13:**
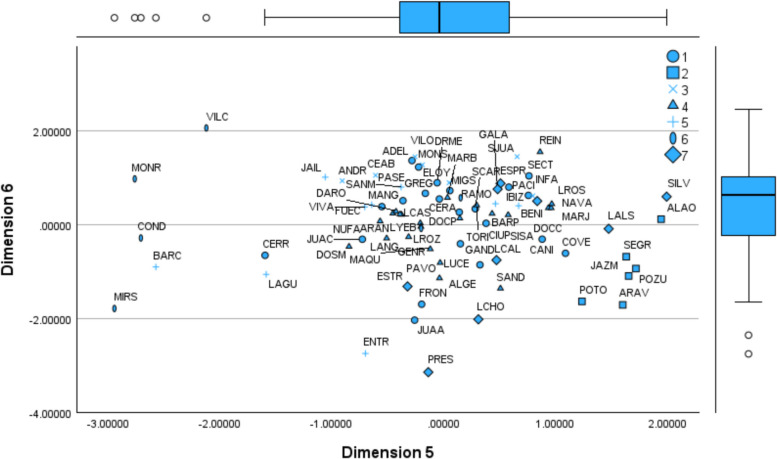
Common Map. Cluster Analysis (Factors 5 and 6)

BENI belongs to cluster five alongside BARC and JAIL, yet it is mapped farther away from its peers. Centres in cluster five share similar output levels and infrastructure costs (D), but BENI employs twice as many staff (AB-medical and C-admin) as comparable centres. For instance, BENI has a significantly larger nursing team yet administrates relatively few vaccines. Factor 3 identifies staff efficiency in providing services, where BENI performs poorly. Given its workforce size, it should be expected to generate twice the output regarding patients served, prescriptions and vaccines administered. The common map for dimension three highlights BENI as a maverick, exhibiting characteristics similar to centres with half its staff, such as BARC, yet belonging to the same cluster. REIN is part of cluster four, along with ARRM, BARP, DARO, MARB, NAVA, NUFA, SISA and TORI. However, for dimension 7, REIN is notably different from its cluster members (see Fig. 9, Appendix 5). REIN has a minimal workforce yet maintain relatively high operating costs (D). Despite this, it records the lowest unitary prescription costs within the same cluster, whereas the others range between 167 (NAVA) and 199 (TORI). With only two administrative employees, REIN sustains a high level of activity, including visits and the lowest prescription costs within its cluster, making it an extreme case —highly efficient in dimension 7 but distinctly different from its peers (maverick).

Inefficient units typically observe their peers to determine a course of action, following the most efficient benchmark. However, this is only relevant if the extreme case influences the efficiency of others and shares the same cluster, implying a similar production function and operational patterns.

Further analysis could provide managerial insights into why centres in clusters four and five perform worse overall (dimension 1) compared to cluster six. However, this falls beyond the scope of this paper.

## Conclusions

This study evaluates the efficiency of primary healthcare centres in Madrid (2018) before the pandemic, using DEA Visualisation for the first time in health research. Traditional DEA benchmarks DMUs based on selected inputs and outputs, often without considering specialisation effects—where inefficiency may stem from diverse activities rather than poor performance.

Results show that overall efficient centres (ABCD123) prescribe more generic drugs (dimension 1), reducing costs for both the centres and the public health system. In contrast, inefficient centres (e.g., REIN, NAVA, VIVA) prescribe costlier specific drugs, possibly due to hidden commissions, requiring further investigation. These centres have low staffing, and higher prescription costs versus highly efficient centres, i.e., MONR, COND and MIRS (dimension 1).

REIN is an extreme case: despite having four to five times fewer doctors, it incurs significantly higher prescription costs suggesting specific rather than generic prescribed drugs, burdening the system. It is super-efficient in non-medical staff (dimension 7) but faces overcapacity in infrastructure (dimension 4)—evidenced by similar visit numbers but lower costs in RAMO. REIN could improve efficiency by absorbing more patients and reallocating medical/admin staff while maintaining non-medical efficiency.

Several centres exhibit overcapacity in generating medical appointments (dimension 4), notably VILO, JAZM, ARAV, POTO, SEGR, and MIRS, all positioned at the top of the map. These centres have sufficient staff and infrastructure to accommodate more visits, meaning efficiency could improve by absorbing a larger population and better-utilising resources.

Conversely, BENI, appearing at the bottom of dimension 4, seems highly efficient. However, this is due to its large workforce (doctors, nurses, and admin staff), making it an extreme case. Reducing BENI’s staff by half or more could create a structure similar to JAIL—both in cluster five—while still maintaining normal activity and absorbing infrastructure costs efficiently.

The results highlight centres that efficiently manage nursing activities (vaccination) have better infrastructure utilisation. POTO and MIRS (bottom of dimension 2) deliver as many vaccines as ELOY, ESTR, INFA, and PRES, despite having lower infrastructure costs and fewer nurses. To improve efficiency, managers should analyse internal procedures, team coordination, and best practices in these high-performing centres to identify strategies that can be adopted by less efficient ones.

DEA Visualisation reveals that centres specialising in preventive medicine—focusing on practices other than drug prescriptions—tend to reduce unitary prescription costs. Centres such as MIRS, MONR, and COND merit further investigation to understand how nurse-led services can prevent illnesses, thereby reducing reliance on prescriptions. For example, promoting a plant-based diet can lower inflammation and decrease chronic conditions, resulting in reduced long-term healthcare costs [66, 67]. These findings align with previous research [68]. Analysing the contextual factors of these centres could further validate these results and clarify their broader implications.

DEA Visualisation identified two extreme cases —REIN and BENI—mavericks rather than outliers due to their isolation within the same cluster. REIN, with the lowest staff levels, was technically efficient across input–output combinations except for high infrastructure costs. However, it showed inefficiency in generic drug prescriptions, with unit costs comparable to centres with three times more doctors, raising concerns about potential pharmaceutical incentives influencing prescriptions. BENI, in contrast, was overstaffed relative to its service levels (visits, vaccines), highlighting inefficiency in resource allocation. These cases suggest staffing imbalances and potential prescription biases, warranting further investigation.

The results show that no centre is understaffed, and all have significant infrastructure investments, though often underutilised. There is no clear correlation between staff levels, capacity, and services provided—some centres have low workloads but high infrastructure costs, while others handle high visit and vaccine numbers efficiently. The mismatch between assigned populations and available resources leads to overloaded or underutilised centres. A review of service distribution is necessary, considering nearby hospitals and their specialisations, as primary care centres serve as gatekeepers before hospital referrals.

Further investigation is needed on the role of admin staff in efficiency, as their tasks are linked to appointments but lack a direct output in the literature. These non-medical employees represent fixed costs for certain centres, impacting overall efficiency. Initially, patient satisfaction was considered a desirable DEA output, but using percentages can create biases, especially disadvantaging smaller or older centres. Facility appearance, often linked to marketing rather than service quality, may lead to misclassification as inefficiency. To improve analysis, we will control for patient satisfaction and incorporate age groups, particularly infants and older adults, as their healthcare needs—such as vaccinations and chronic care—could significantly impact efficiency rankings.

For larger health datasets with multiple input and output variables, feature selection models, including machine learning, may better identify relevant variables for primary care efficiency. Given the high number of DEA specifications (four inputs, three outputs), feature selection can help reduce models, which can then, be mapped using cluster analysis and ProFit for better visual interpretation.

New primary care trends, such as online consultations and restricting emergency rooms to real emergencies, contribute to resource optimisation in both primary care and hospitals. Future research should assess how these changes impact congestion reduction (undesirable output) and cost savings.

## Data Availability

The data are not public.

## Appendix 1

**Table 5:** DEA Specifications models

DEA Specifications	Inputs	Outputs
ABCD123 Traditional DEA model	ABCD (Number of Doctors, Nurses& Admin Staff, Infrastructure Costs)	123 (Visits, Prescription Cost, Vaccines)
A1, A2, A3 B1, B2, B3 C1, C2, C3 D1, D2, D3 A12, A13, A23, A123 B12, B13, B23, B123 C12, C13, C23, C123 D12, D13, D23, D123	A (Number of Doctors) B (Number of Nurses) C (Admin Staff) D (Infrastructure Costs)	1 (Visits) 2 (Prescription Cost) 3 (Vaccines) 12 (Visits, Prescription Cost) 13 (Visits, Vaccines) 23 (Prescription Cost, Vaccines) 123 (Visits, Prescription Cost, Vaccines)
AB1, AB2, AB3, AB12, AB13, AB23, AB123 AC1, AC2, AC3, AC12, AC13, AC23, AC123 AD1, AD2, AD3, AD12, AD13, AD23, AD123 BC1, BC2, BC3, BC12, BC13, BC23, BC123, BD1, BD2, BD3, BD12, BD13, BD23, BD123CD1, CD2, CD3, CD12, CD13, CD23, CD123	AB (Number of Doctors & Nurses) AC (Number of Doctors & Admin) AD (Number of Doctors, Infrastructure Costs) BC (Number of Nurses & Admin)BD (Number of Nurses, Infrastructure Costs)CD (Admin, Infrastructure Costs)	
ABC1, ABC2, ABC3, ABC12, ABC13, ABC23, ABC123 ABD1, ABD2, ABD3, ABD12, ABD13, ABD23, ABD123 ACD1, ACD2, ACD3, ACD12, ACD13, ACD23, ACD123 BCD1, BCD2, BCD3, BCD12, BCD13, BCD23, BCD123	ABC (Number of Doctors, Nurses & Admin) ABD (Number of Doctors & Nurses, Infrastructure Costs) ACD (Number of Doctors & Admin, Infrastructure Costs) BCD (Number of Nurses & Admin, Infrastructure Costs)	
ABCD1, ABCD2, ABCD3, ABCD12, ABCD13, ABCD23	ABCD (Number of Doctors, Nurses & Admin Staff, Infrastructure Costs)	

This table presents the traditional DEA model—i.e., including all inputs (A, B, C, D) and outputs (1, 2, 3)—along with the alternative specifications based on their combinations. We estimate the efficiencies for 105 models (DEA specifications)

**Table 6:** Health Centres Sample Acronyms

Health-Centre	Acronym	Health-Centre	Acronym	Health-Centre	Acronym
Adelfas	ADEL	Fronteras	FRON	María Jesús Hereza	MARJ
Alameda de Osuna	ALAO	Fuencarral	FUEC	Miguel Servet	MIGS
Algete	ALGE	Galapagar	GALA	Mirasierra	MIRS
Andrés Mellado	ANDR	Gandhi	GAND	Monterrozas	MONR
Aranjuez	ARAN	General Ricardos	GENR	Montesa	MONS
Aravaca	ARAV	Goya	GOYA	Navalcarnero	NAVA
Arroyo Medialegua	ARRM	Gregorio Marañón	GREG	Nuestra Señora de Fátima	NUFA
Barcelona	BARC	Guayaba	GUAY	Pacífico	PACI
Barrio del Pilar	BARP	Ibiza	IBIZ	Paseo Imperial	PASE
Benita de Ávila	BENI	Infanta Mercedes	INFA	Pavones	PAVO
Canal de Panamá	CANA	Jaime Vera—Leganés	JAIL	Potosí	POTO
Canillejas	CANI	Jazmín	JAZM	Pozuelo—Estación	POZU
Cea Bermúdez	CEAB	Juan de Austria	JUAA	Presentación Sabio	PRES
Cerro Almodóvar	CERR	Juan de la Cierva	JUAC	Ramón y Cajal	RAMO
Cerro del Aire	CERA	La Chopera	LCHO	Reina Victoria	REIN
Ciudad Periodistas	CIUP	Las Águilas	LAGU	San Andrés	SAND
Collado Villalba Estación	COVE	Las Calesas	LCAL	San Carlos	SCAR
Condes de Barcelona	COND	Las Rozas—El Abajon	LROZ	San Juan de la Cruz	SJUA
Daroca	DARO	Los Alpes	LALS	Sánchez Morate	SANM
Doctor Cirajas	DOCC	Los Ángeles	LANG	Santa Isabel	SISA
Doctor Pedro Laín Entralgo	DOCP	Los Castillos	LCAS	Sector III	SECT
Dos de Mayo	DOSM	Los Rosales	LROS	Segre	SEGR
Dr. Castroviejo	DRCA	Los Yébenes	LYEB	Silvano	SILV
Dr. Mendiguchía Carriche	DRME	Lucero	LUCE	Torito	TORI
Eloy Gonzalo	ELOY	M. Angeles López Gómez	MANG	Villa de Vallecas	VIVA
Entrevías	ENTR	Maqueda	MAQU	Villanueva de la Cañada	VILC
Espronceda	ESPR	Mar Báltico	MARB	Villaviciosa de Odón	VILO
Estrecho de Corea	ESTR				

## Appendix 2

**Table 7:** DEA Efficiencies (13 DEA Specifications)

	ABCD123	ABC123	ABC13	ABC1	BC3	BD123	BD2	BD3	A12	A13	B13	C123	C12
ANDR	133%	149%	287%	327%	287%	133%	136%	147%	154%	287%	287%	153%	644%
ARRM	100%	100%	129%	167%	129%	100%	109%	100%	105%	133%	134%	100%	204%
BARP	100%	103%	120%	128%	120%	100%	115%	105%	107%	123%	120%	103%	245%
BENI	100%	120%	248%	269%	248%	100%	100%	100%	120%	248%	248%	120%	564%
CEAB	128%	142%	272%	272%	291%	128%	141%	138%	144%	274%	272%	148%	398%
DOCP	122%	135%	224%	224%	227%	122%	134%	136%	136%	224%	224%	135%	586%
DRME	125%	132%	215%	226%	215%	125%	137%	132%	135%	217%	219%	132%	437%
ELOY	141%	151%	221%	221%	224%	141%	162%	157%	153%	221%	221%	152%	602%
ESPR	134%	151%	180%	209%	180%	134%	179%	134%	159%	180%	180%	151%	881%
ESTR	127%	137%	202%	202%	239%	127%	144%	165%	137%	202%	202%	137%	493%
GALA	134%	153%	174%	174%	182%	134%	187%	147%	153%	174%	174%	153%	571%
IBIZ	121%	140%	187%	217%	187%	121%	158%	121%	149%	187%	187%	140%	538%
INFA	139%	162%	226%	226%	232%	139%	175%	156%	163%	226%	226%	163%	650%
JAIL	100%	101%	231%	241%	231%	100%	100%	100%	101%	232%	231%	105%	284%
JAZM	124%	168%	231%	339%	231%	124%	173%	124%	181%	238%	237%	168%	485%
LAGU	100%	100%	185%	185%	197%	100%	100%	131%	100%	185%	185%	100%	386%
LCAL	125%	144%	210%	210%	236%	125%	156%	153%	145%	210%	210%	144%	524%
LALS	122%	153%	166%	166%	173%	122%	177%	142%	154%	168%	166%	155%	476%
LANG	102%	103%	153%	153%	155%	102%	112%	118%	103%	153%	153%	103%	586%
LROS	122%	146%	164%	164%	167%	122%	180%	131%	146%	164%	164%	146%	654%
LYEB	102%	103%	139%	155%	139%	102%	117%	108%	107%	139%	139%	103%	741%
MANG	103%	105%	189%	190%	189%	103%	106%	112%	105%	191%	191%	106%	232%
MARB	100%	100%	100%	100%	100%	100%	129%	100%	100%	100%	100%	100%	281%
MIRS	100%	116%	116%	199%	116%	100%	222%	100%	191%	120%	117%	116%	381%
MONS	141%	154%	265%	285%	265%	141%	148%	150%	159%	265%	265%	154%	1018%
NAVA	100%	100%	100%	100%	100%	100%	103%	100%	100%	100%	100%	100%	146%
NUFA	101%	101%	122%	128%	122%	101%	115%	114%	104%	126%	124%	101%	303%
PACI	121%	132%	173%	204%	175%	124%	144%	124%	136%	173%	175%	136%	555%
PASE	126%	127%	142%	142%	142%	126%	158%	129%	127%	142%	142%	127%	499%
POTO	100%	134%	181%	282%	181%	100%	143%	100%	143%	186%	186%	134%	311%
RAMO	100%	108%	184%	209%	184%	100%	105%	100%	113%	191%	190%	108%	297%
REIN	100%	100%	100%	100%	100%	100%	100%	100%	100%	100%	100%	100%	100%
SJUA	135%	153%	225%	256%	225%	135%	160%	136%	159%	229%	225%	156%	573%
SANM	100%	102%	202%	202%	203%	100%	105%	102%	102%	203%	204%	104%	197%
SISA	102%	110%	146%	154%	146%	102%	117%	109%	111%	146%	146%	111%	293%
SEGR	121%	166%	231%	323%	231%	121%	171%	121%	178%	238%	234%	166%	477%
TORI	100%	100%	140%	154%	140%	100%	109%	100%	105%	140%	140%	101%	378%
VIVA	100%	100%	158%	158%	161%	100%	100%	109%	100%	182%	181%	100%	160%
VILC	129%	133%	133%	133%	134%	129%	221%	131%	133%	133%	133%	133%	593%
VILO	140%	149%	236%	287%	236%	140%	154%	140%	160%	247%	238%	150%	418%
**Frontier**	14	6	3	3	3	14	5	10	5	3	3	6	1

This table shows a sample of 13 out of the 105 DEA specifications results to highlight significant differences that arise when selecting different combinations of inputs and outputs for a subset of DMUs. A unit that is on the efficiency frontier in the traditional model (ABCD123) may become inefficient whenone or more inputs or outputs are removed. The "Frontier" row indicates is the number of efficient health centres for each specification

## Appendix 3

**Table 8:** Rotated Component Matrix: Factor loadings^a^

	**PC1**	**PC2**	**PC3**	**PC4**	**PC5**	**PC6**	**PC7**
ABCD123	0.391	0.743					
ABCD12	0.391	0.743					
ABCD13		0.748					
ABCD23	0.355	0.874					
ABCD1	0.672		0.353	0.601			
ABCD2	0.974						
ABCD3		0.933					
ABC123	0.695	0.410	0.366		0.418		
ABC12	0.895						
ABC13			0.938				
ABC23	0.692	0.453			0.423		
ABC1			0.902				
ABC2	0.981						
ABC3		0.438	0.890				
ABD123	0.386	0.745					
ABD12	0.756			0.504			
ABD13		0.748					
ABD23		0.880					
ABD1	0.671		0.351	0.604			
ABD2	0.974						
ABD3	0.050	0.933					
ACD123	0.413	0.711				0.357	
ACD12	0.759			0.489			
ACD13		0.717	0.369			0.356	
ACD23	0.368	0.851					
ACD1	0.674		0.366	0.593			
ACD2	0.974						
ACD3	0.056	0.930					
BCD123	0.390	0.742					
BCD12	0.757			0.502			
BCD13		0.746					
BCD23	0.354	0.874					
BCD1	0.671		0.354	0.602			
BCD2	0.974						
BCD3		0.932					
AB123	0.695	0.400	0.370		0.422		
AB12	0.695	0.400	0.370		0.422		
AB13			0.942				
AB23	0.692	0.445			0.426		
AB1			0.904				
AB2	0.981						
AB3		0.421	0.897				
AC123	0.695	0.407	0.373		0.413		
AC12	0.890						
AC13			0.938				
AC23	0.692	0.451			0.420		
AC1			0.902				
AC2	0.982						
AC3		0.438	0.891				
AD123	0.410	0.705				0.360	
AD12	0.760			0.497			
AD13		0.707	0.367			0.364	
AD23	0.361	0.855					
AD1	0.674		0.362	0.602			
AD2	0.974						
AD3		0.927					
BC123	0.694	0.409	0.371		0.416		
BC12	0.894						
BC13			0.937				
BC23	0.691	0.452			0.421		
BC1			0.902				
BC2	0.981						
BC3		0.439	0.890				
BD123	0.385	0.744					
BD12	0.755			0.506			
BD13		0.747					
BD23		0.879					
BD1	0.670		0.352	0.605			
BD2	0.975						
BD3		0.932					
A123	0.700	0.390	0.377		0.413		
A12	0.887						
A13			0.943				
A23	0.696	0.439			0.420		
A1			0.903				
A2	0.982						
A3		0.417	0.900				
B123	0.694	0.399	0.377		0.419		
B12	0.893						
B13			0.942				
B23	0.690	0.444			0.424		
B1			0.905				
B2	0.981						
B3		0.420	0.898				
C123	0.692	0.400	0.384		0.412		
C12		0.379					0.708
C13			0.937				
C23	0.690	0.444	0.355		0.422		
C1	0.268		0.902				
C2	0.980						
C3		0.437	0.891				
D123	0.382	0.610				0.432	
D12	0.729			0.535			
D13		0.601	0.361	0.369			
D23		0.742					-0.415
D1	0.642			0.625			
D2	0.934						
D3		0.790					-0.485

^a^ Figures lower than 0.4 in absolute value have been removed

This table shows the loadings of each DEA specification on the extracted factors. This information it is possible allows us to identify which specifications are more relevant—that is, which ones contribute more strongly to each factor

## Appendix 4

**Table 9:** ProFit Normalised Regression Coefficients

**DEA Specifications**							
ABCD123	0.394	0.748	0.286	0.225	0.125	0.349	0.128
ABCD12	0.394	0.748	0.286	0.225	0.125	0.349	0.128
ABCD13	0.274	0.754	0.339	0.300	-0.021	0.334	0.197
ABCD23	0.361	0.889	0.177	-0.008	0.195	0.093	0.015
ABCD1	0.674	0.208	0.354	0.602	0.031	0.058	0.105
ABCD2	0.975	0.157	-0.136	0.030	-0.044	0.046	0.036
ABCD3	0.051	0.936	0.211	-0.018	-0.068	-0.264	0.039
ABC123	0.697	0.411	0.367	0.156	0.420	0.086	0.051
ABC12	0.906	0.139	0.269	0.223	0.195	0.000	0.007
ABC13	0.000	0.308	0.939	0.055	0.044	0.130	0.037
ABC23	0.694	0.454	0.340	0.121	0.424	0.027	0.038
ABC1	0.266	0.022	0.905	0.300	0.130	0.039	0.038
ABC2	0.982	0.145	-0.072	0.030	-0.034	0.057	0.058
ABC3	-0.045	0.439	0.892	-0.027	0.017	-0.088	-0.017
ABD123	0.389	0.750	0.288	0.228	0.119	0.348	0.128
ABD12	0.762	0.214	0.303	0.508	0.131	0.057	0.055
ABD13	0.271	0.755	0.337	0.302	-0.022	0.334	0.198
ABD23	0.354	0.894	0.180	-0.009	0.189	0.083	0.012
ABD1	0.672	0.207	0.352	0.605	0.030	0.058	0.104
ABD2	0.976	0.160	-0.128	0.036	-0.042	0.038	0.033
ABD3	0.050	0.936	0.210	-0.017	-0.069	-0.264	0.039
ACD123	0.417	0.717	0.313	0.225	0.169	0.361	0.073
ACD12	0.766	0.207	0.312	0.494	0.154	0.067	0.026
ACD13	0.296	0.727	0.374	0.311	0.023	0.361	0.129
ACD23	0.375	0.867	0.191	-0.001	0.245	0.105	-0.029
ACD1	0.677	0.201	0.368	0.595	0.051	0.069	0.069
ACD2	0.976	0.154	-0.140	0.027	-0.040	0.046	0.027
ACD3	0.057	0.936	0.260	-0.002	-0.044	-0.225	-0.027
BCD123	0.393	0.747	0.285	0.227	0.126	0.350	0.128
BCD12	0.763	0.215	0.301	0.506	0.136	0.060	0.056
BCD13	0.272	0.753	0.339	0.303	-0.021	0.335	0.197
BCD23	0.360	0.889	0.177	-0.005	0.196	0.095	0.015
BCD1	0.672	0.208	0.354	0.603	0.030	0.058	0.105
BCD2	0.975	0.156	-0.139	0.031	-0.042	0.048	0.036
BCD3	0.049	0.936	0.213	-0.014	-0.072	-0.263	0.039
AB123	0.698	0.402	0.371	0.160	0.423	0.080	0.048
AB12	0.698	0.402	0.371	0.160	0.423	0.080	0.048
AB13	-0.008	0.291	0.944	0.063	0.043	0.131	0.021
AB23	0.694	0.447	0.344	0.124	0.428	0.018	0.035
AB1	0.259	0.014	0.907	0.302	0.127	0.040	0.026
AB2	0.982	0.148	-0.070	0.029	-0.033	0.050	0.059
AB3	-0.056	0.422	0.899	-0.020	0.013	-0.087	-0.036
AC123	0.697	0.409	0.374	0.153	0.415	0.098	0.035
AC12	0.901	0.140	0.280	0.224	0.198	0.010	-0.005
AC13	0.006	0.305	0.940	0.054	0.045	0.133	0.034
AC23	0.695	0.453	0.345	0.117	0.422	0.040	0.021
AC1	0.268	0.022	0.905	0.298	0.131	0.040	0.036
AC2	0.983	0.143	-0.069	0.031	-0.030	0.060	0.048
AC3	-0.039	0.439	0.893	-0.032	0.020	-0.085	-0.023
AD123	0.414	0.712	0.314	0.238	0.171	0.364	0.066
AD12	0.767	0.195	0.310	0.502	0.148	0.062	0.019
AD13	0.290	0.720	0.374	0.327	0.025	0.371	0.110
AD23	0.368	0.871	0.197	0.005	0.240	0.090	-0.038
AD1	0.677	0.186	0.363	0.605	0.046	0.069	0.053
AD2	0.977	0.160	-0.128	0.037	-0.033	0.033	0.022
AD3	0.055	0.935	0.264	0.007	-0.043	-0.221	-0.037
BC123	0.696	0.410	0.372	0.156	0.418	0.087	0.049
BC12	0.905	0.138	0.273	0.223	0.194	0.001	0.005
BC13	0.000	0.308	0.939	0.056	0.044	0.130	0.037
BC23	0.693	0.454	0.345	0.121	0.422	0.028	0.036
BC1	0.266	0.022	0.905	0.300	0.131	0.039	0.038
BC2	0.983	0.143	-0.072	0.031	-0.033	0.059	0.056
BC3	-0.045	0.439	0.892	-0.026	0.017	-0.088	-0.017
BD123	0.387	0.749	0.288	0.231	0.119	0.349	0.129
BD12	0.761	0.213	0.303	0.510	0.131	0.058	0.054
BD13	-0.009	0.291	0.944	0.064	0.043	0.132	0.021
BD23	0.351	0.894	0.184	-0.009	0.187	0.086	0.010
BD1	0.671	0.207	0.353	0.606	0.029	0.058	0.104
BD2	0.976	0.156	-0.128	0.035	-0.041	0.041	0.031
BD3	0.046	0.936	0.215	-0.016	-0.073	-0.263	0.037
A123	0.703	0.392	0.379	0.168	0.414	0.092	0.026
A12	0.898	0.136	0.283	0.232	0.201	0.008	-0.008
A13	0.006	0.283	0.945	0.074	0.050	0.136	0.013
A23	0.699	0.440	0.351	0.129	0.421	0.025	0.011
A1	0.263	0.013	0.905	0.304	0.129	0.041	0.022
A2	0.984	0.150	-0.065	0.033	-0.025	0.046	0.048
A3	-0.042	0.418	0.902	-0.014	0.021	-0.083	-0.048
B123	0.696	0.400	0.378	0.160	0.421	0.083	0.045
B12	0.904	0.134	0.277	0.223	0.195	0.000	0.004
B13	-0.009	0.291	0.944	0.064	0.043	0.132	0.021
B23	0.693	0.446	0.351	0.124	0.426	0.021	0.032
B1	0.258	0.014	0.907	0.302	0.127	0.040	0.026
B2	0.983	0.145	-0.069	0.029	-0.033	0.054	0.057
B3	-0.058	0.421	0.900	-0.020	0.012	-0.086	-0.037
C123	0.696	0.402	0.386	0.142	0.415	0.109	0.022
C12	0.328	0.432	0.163	0.135	0.089	0.044	0.807
C13	0.010	0.302	0.940	0.049	0.052	0.139	0.033
C23	0.693	0.446	0.357	0.105	0.424	0.051	0.005
C1	0.269	0.022	0.905	0.297	0.134	0.042	0.036
C2	0.985	0.131	-0.079	0.015	-0.008	0.077	0.025
C3	-0.034	0.438	0.893	-0.037	0.029	-0.079	-0.025
D123	0.386	0.618	0.258	0.310	0.252	0.437	-0.228
D12	0.736	0.138	0.258	0.540	0.180	0.119	-0.187
D13	0.251	0.615	0.370	0.378	0.091	0.480	-0.202
D23	0.281	0.760	0.098	0.080	0.336	0.183	-0.425
D1	0.647	0.106	0.349	0.629	0.069	0.138	-0.167
D2	0.942	0.062	-0.237	0.047	0.007	0.085	-0.209
D3	-0.083	0.816	0.263	0.018	0.009	-0.080	-0.501
Max	0.985	0.936	0.945	0.629	0.428	0.480	0.807
Min	-0.083	0.013	-0.237	-0.037	-0.073	-0.264	-0.501
Std. Dev	0.348	0.285	0.327	0.187	0.154	0.149	0.128

This table provides the position of each DEA specification on the Common Map, allowing them to be located on the same map as the DMUs. This facilitates the identification of the information captured and to name the extracted factors

## Appendix 5

Figures 8 and 9 show the health centres across the extracted factors 5, 6, 1, and 7.

## Appendix 6

Figures 10 and 11 show the 105 DEA Specifications across the extracted factors 5, 6, 1, and 7.

## Appendix 7

Figures 12 and 13 show the health centres classified in clusters across the extracted factors 1, 2, 5, and 6.
